# Interface Tuning between Two Connecting Bulk Heterojunctions in Small Molecule Bilayer Ternary Solar Cells

**DOI:** 10.3390/ma13214833

**Published:** 2020-10-29

**Authors:** Qi Jiang, Yingjie Xing

**Affiliations:** Key Laboratory for the Physics and Chemistry of Nanodevices, Beijing Key Laboratory of Quantum Devices, and Department of Electronics, Peking University, Beijing 100871, China; 1701213869@pku.edu.cn

**Keywords:** organic solar cell, bilayer ternary structure, interface, carrier recombination, C60 molecular monolayer

## Abstract

Bilayer ternary solar cells are a kind of novel organic photovoltaic device with a triple-component active layer but are different from the ternary bulk heterojunction (BHJ) blend. Two binary BHJs with a common acceptor (or donor) are deposited sequentially in this kind of device. Here, we study the fabrication and optimization of bilayer ternary solar cells using metal phthalocyanine donors and fullerene acceptor. The device power conversion efficiency (PCE) shows a significant dependence on the interface between the two binary BHJs. The interface formed by stacking two BHJs directly demonstrates severe restrictions on the device efficiency. We find that the photovoltaic performance of bilayer ternary cells can be improved by inserting a C60 molecular monolayer between the two binary BHJs. The effect of the C60 interfacial layer on charge transport is analyzed based on their transport characteristics under negative bias. A relationship between the C60 interfacial layer and recombination under illumination is discussed. This work reveals a particular influence due to the interface facing three materials in organic solar cells.

## 1. Introduction

**Figure 1:**
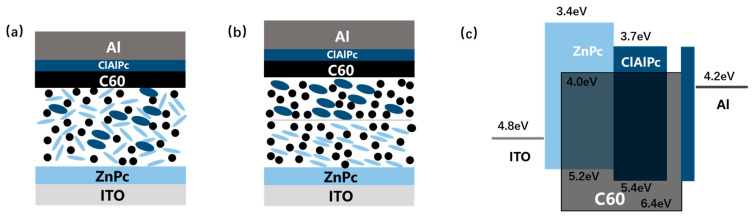
Schematically demonstration of (**a**) ternary bulk heterojunction (BHJ) structure and (**b**) bilayer ternary structure. (**c**) Band diagram of ZnPc:C60/ClAlPc:C60 binary ternary structure. Energy level values are adopted from Ref. [17,18]. Light blue ellipse, dark blue ellipse, and black dot stand for ZnPc, ClAlPc, and C60 molecule, respectively.

An efficiency higher than 10% has been achieved in organic solar cells by employing novel polymers with higher mobility and a wider optical absorption window in the last decade [1,2]. Binary bulk heterojunction (BHJ), which is a molecular scale mixture containing a donor and an acceptor, is used as the device structure in all these devices. The record efficiency of binary BHJ cells (18.2%), which is higher than those of tandem cells (17.3%) and modified planar heterojunction cells (4.0%), indicates that BHJ is still the most promising structure in organic solar cells [3,4,5]. However, it becomes more and more difficult to further enhance the photovoltaic performance by synthesizing more ideal polymers [2,6]. Recently, some progress has been obtained in a different way, by searching for novel organic semiconductors. An additional component with complementary absorption spectra (donor D2 for donor D1 and acceptor A, or acceptor A2 for donor D and acceptor A1) was blended with a binary BHJ to form a ternary BHJ (shown in Figure 1a). Some properties of photovoltaic cells, e.g., short-circuit current density (Jsc), open-circuit voltage (Voc), fill factor (FF), and the thermal stability can be improved by using triple components together after carefully optimizing the morphology and ratio of ternary BHJ [7,8]. A record efficiency of more than 16% is reported by blending one nonfullerene acceptor into the mixture of a polymer and a fullerene derivative, and in a ternary BHJ device composing one polymer donor and two nonfullerene acceptors [9,10]. Detailed study reveals dedicated roles of the third component in ternary BHJ devices, including absorbing complementary light, tuning the molecular energy levels, and adjusting the blend morphology [8]. Nonetheless, because the miscibility and crystallization in ternary systems are quite different from those in the host binary BHJs, due to the addition of the third component, the high efficiency in ternary BHJ devices is often obtained through trial and error rather than theoretical estimation. It is found that the third component brings some unknown variations in ternary BHJs, e.g., the location of the third component in the ternary blend (both in the nanoscale morphology and in the bulk), which does not emerge in binary BHJ [8]. Furthermore, the working mechanism of ternary photovoltaic device may change partly due to the distribution style of the third component, including whether it is embedded in the donor or acceptor phase, located between the donor and acceptor phases, or co-crystallized with the donor or acceptor moiety. For example, a thoroughly mixed microstructure is beneficial to the energy cascade model, whereas for the alloy model, two components in the alloy phase should have a great enough miscibility [8,11,12]. The status in an actual ternary BHJ may be more complex because the ternary blend can comprise different locations of the third component depending on the varied ratio under different conditions. It is thus summarized, that the mechanism of ternary BHJ devices is material system dependent [8,13,14].

Lacking an effective guideline for adding an additional third component encourages other attempts to assemble the ternary system. Novel device architecture, namely bilayer ternary structure, is particularly designed to maintain the benefits of both the wide absorption window of the ternary system and the simplified binary microstructure [15,16]. Such a bilayer ternary structure contains two binary BHJs (D1:A and D2:A, or D:A1 and D:A2) deposited in series, resulting in a D1:A/D2:A (or D:A1/D:A2) active layer. In a typical bilayer ternary structure, two consequently prepared binary BHJs are segregated by a sharp interface, which is different from the thin transparent electrode between two subcells in a tandem device [1]. Figure 1b shows the structure of D1:A/D2:A bilayer ternary device schematically. Besides the common mechanism of exciton generation and dissociation in a single binary BHJ, some roles are proposed for governing charge transport across the interface in a bilayer ternary active layer as following: the lowest unoccupied molecular orbital (LUMO) of the same acceptor (A) in both binary BHJs acts as a continuous route for electron transport, whereas for hole transport, the highest occupied molecular orbital (HOMO) of two polymers should have a suitable step to assist the hole extraction. A supposed band diagram of bilayer ternary structure is drawn in Figure 1c schematically. The above mechanism defines a different working model from the ternary BHJ device. In bilayer ternary devices, Voc is restricted by the smallest gap between the HOMO and LUMO of triple components, and Jsc is decided by both the complementary absorption and the bilayer transport route. There are few up to date demonstrations of polymer bilayer ternary devices [15,16]. Ghasemi et al. use a low-bandgap polymer, which is hard-to-dissolve at room temperature, to form a stable bottom BHJ film with a fullerene derivative. Then a solution containing a middle-bandgap polymer and the same fullerene derivative is spin-coated on it. The bilayer ternary device shows an efficiency improvement from 6.39% to 6.73% compared to the host binary BHJ device [16]. Colberts et al. developed a method to form a thin BHJ film on a water surface by spontaneous spreading of polymer–fullerene blends. Bilayer ternary structure is produced by transferring a floating BHJ layer on a previously spin-coated bottom binary BHJ layer. The bilayer ternary device shows an efficiency of 5.9%, which is higher than the 5.1% efficiency of the host binary BHJ cell [15]. These results show the potential of the bilayer ternary structure as an alternative method to improve binary BHJ organic solar cells.

It is reasonable to study whether the charge transport inside an actual bilayer ternary device occurs similarly to the above model exactly. In fact, an electron barrier is suggested in bilayer ternary devices, fabricated by transferring, which hints a device-dependent defect at the interface between two binary BHJs [15]. This barrier effect appears in bilayer ternary devices prepared with different polymers, suggesting that the trial and error attempt by material variation is not capable of further optimization for these kinds of devices. Even after detailed investigations of the bilayer ternary device, it is still clear where and why the barrier appears in this kind of devices [15]. There is no clue how to eliminate this problem in the literature.

Small molecule solar cells are generally fabricated by thermal deposition in a vacuum chamber. Although the efficiency of small molecule cells is usually lower than that in the polymer devices, small molecule cells are a proper candidate to study the working mechanism of organic photovoltaic devices, because the device factors can be varied controllably and accurately by adjusting the evaporation conditions, e.g., deposition rate and time [1,19,20]. We fabricate small molecule bilayer ternary devices in the structure of D1:A/D2:A and investigate their unideal photovoltaic behavior. We find that the interface between two binary BHJs is the key factor influencing the performance of the bilayer ternary device. In contrast to the general assumption in the device model of bilayer ternary devices, a sharp interface between two BHJs does not mean an efficient transport route for electrons. Our result reveals that the interface in bilayer ternary structure cannot be simply treated as a copy in binary BHJ device, indicating a difference between binary BHJ devices and bilayer ternary devices.

## 2. Materials and Methods

Cleaned indium tin oxide (ITO) glass is used as the substrate in all devices. Two kinds of small molecule, zinc phthalocyanine (ZnPc, Yannuo Chem., Beijing, China, purity 99%) and chloroaluminum phthalocyanine (ClAlPc, Yannuo Chem., Beijing, China, purity 99%), are used as the donors. C60 (Yannuo Chem., Beijing, China, purity 99%) is used as the acceptor. Aluminum is used as the cathode metal. These materials are used as received. All devices are fabricated in a commercial vacuum deposition system (ULVAC-KIKO VWR-400M/ERH). No other material is deposited because our deposition system has only four sites for source materials. We use ClAlPc as both donor and cathode buffer material in this experiment. The architecture of bilayer ternary devices is ITO/ZnPc/ZnPc:C60/ClAlPc:C60/C60/ClAlPc/Al. Typical photovoltaic performance is measured in the device. Figure 1b,c shows the device structure and its proposed energy level diagram. ZnPc:C60 and ClAlPc:C60 BHJs have ratios of 1:1 and 1:2, which were chosen after many rounds of optimization. All organic components are deposited onto ITO substrates at a rate of ~0.05 nm/s under a pressure of ~8 × 10^−4^ Pa. Al is deposited at a rate of ~0.5 nm/s under a pressure of ~5 × 10^−3^ Pa. The thicknesses of ZnPc (10 nm)/ZnPc:C60 (20–30 nm)/ClAlPc:C60 (20–30 nm)/C60 (15 nm)/ClAlPc (3 nm)/Al (80 nm) layers are used in this work. The film thickness and deposition rate are monitored in situ using a quartz crystal oscillator. At least three batches of photovoltaic devices are prepared under each condition. The best efficiency in each batch is mentioned in this work. A sunlight simulator (Newport Oriel 91160) is used to illuminate the sample with the power of 100 mW/cm^2^. The current–voltage curves are measured by an electrochemical analyzer in air. Some parameters of the measured device are extracted from the density–voltage (J–V) curves according to a one-diode model [21]. Optical absorption is measured with a UV-Vis-NIR spectrophotometer (Cary 5000, Agilent, Santa Clara, CA, USA) in air.

We find the interface between ZnPc:C60 BHJ and ClAlPc:C60 BHJ has a critical influence on the efficiency of bilayer ternary devices. While maintaining the same conditions for all other parts, three kinds of devices are classified according to different interfacial status. A standard interface is made by simultaneously shutting the heating current of ZnPc and C60. An improved interface is made by shutting the heating current of C60 10 s later than shutting that of ZnPc. A comparable interface is also made by shutting the heating current of ZnPc 10 s later than shutting that of C60. Such an interface fabrication procedure can be repeated in our experiment reliably.

## 3. Results and Discussion

**Figure 2:**
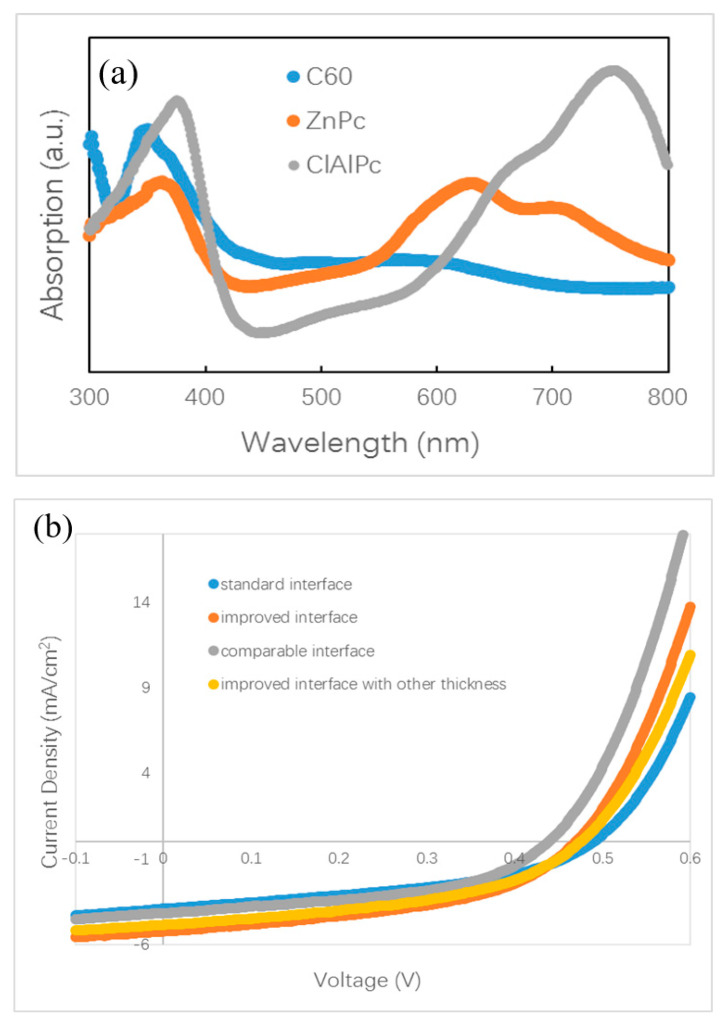

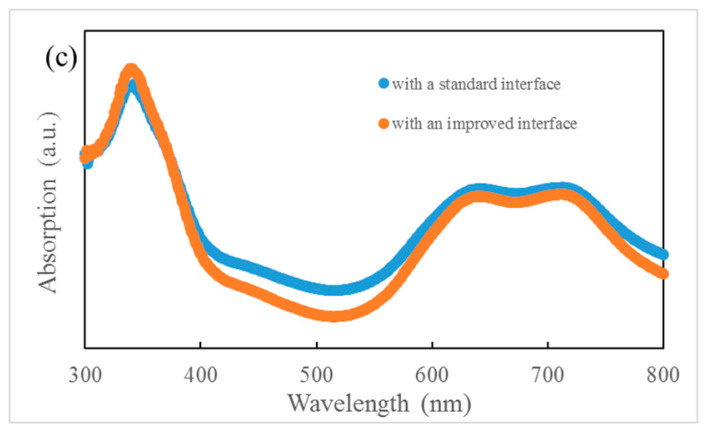
(**a**) Optical absorption spectra of ZnPc, ClAlPc, and C60. (**b**) density–voltage (J–V) curves of ZnPc:C60/ClAlPc:C60 bilayer ternary devices. (**c**) Optical absorption spectra of ZnPc:C60/ClAlPc:C60 bilayer with a standard or improved interface.

There are no small molecule bilayer ternary solar cells reported in the literature. Here, we fabricate and study a small molecule bilayer ternary photovoltaic device for the first time. The designing role for this device is similar to the framework introduced briefly in the above section. We choose two donors, ZnPc and ClAlPc, and one acceptor, C60, to construct the bilayer ternary structure. ZnPc and C60 are a common donor–acceptor pair with optical absorption in the visible band, and ClAlPc has an absorption peak in the near infrared band [22,23]. The optical absorption spectra of these materials are shown in Figure 2a, demonstrating complementary absorption spectra. The bilayer ternary structure (D1:A/D2:A) is prepared by depositing ClAlPc:C60 BHJ behind ZnPc:C60 BHJ. The band diagram of this device is shown in Figure 1c. The arrangement of ClAlPc:C60 BHJ behind ZnPc:C60 BHJ is to create a HOMO cascade for facilitating hole extraction from ClAlPc:C60 BHJ.

**Figure 3:**
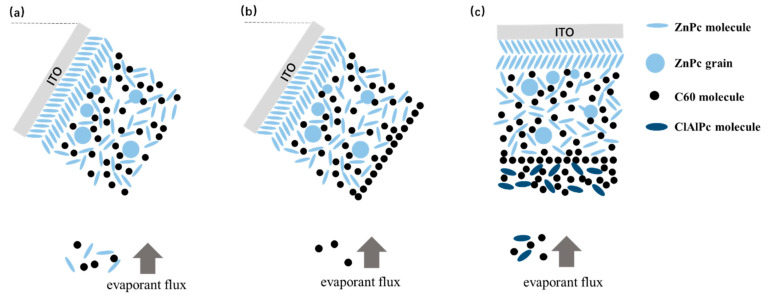
Schematically demonstration of (**a**) oblique deposition of ZnPc/ZnPc:C60, (**b**) deposition of a monolayer of C60 molecules, and (**c**) deposition of ClAlPc:C60 on a horizontal substrate.

The fabrication of bilayer ternary solar cells is based on our previous result of binary BHJ devices. We have already shown that oblique angle deposition is an effective approach to improve the photovoltaic performance of BHJ devices with ZnPc as the donor [22]. Briefly, spontaneous phase segregation occurs in ZnPc:acceptor BHJ when ZnPc/ZnPc:acceptor layers are deposited on an obliquely placed ITO substrate, resulting in enhanced Jsc and efficiency. More details of the oblique angle deposition are described in the Supplementary Information (Figure S1). Figure 3a shows the oblique deposition style schematically. Here, we directly use this oblique angle deposition technique to prepare ZnPc/ZnPc:C60 bilayer firstly. Then the substrate is rotated in situ to the horizontal plane for subsequent deposition of ClAlPc:C60/C60/ClAlPc/Al layers.

Polymer bilayer ternary devices have been fabricated by stacking two binary BHJ layers directly [15,16]. The greatest efficiency for a polymer bilayer ternary device is obtained by optimization of the thicknesses of two binary BHJ layers. It is believed that a balance between the wide optical absorption and the efficient charge transport is approached in optimal polymer bilayer ternary devices. For small molecule bilayer ternary cells, we also optimize the thicknesses of all isolated layers, e.g., ZnPc:C60 BHJ, ClAlPc:C60 BHJ, and ClAlPc layer. We found that the device performance is influenced by not only the thickness of the BHJs but also the thickness of the cathode buffer layer. We also changed other conditions, e.g., the evaporation rate, to adjust the D/A ratio in BHJ. After careful optimization, the conditions were fixed to ZnPc (10 nm)/ZnPc:C60 (30 nm, 1:1)/ClAlPc:C60 (30 nm, 1:2)/C60 (15 nm)/ClAlPc (3 nm)/Al (80 nm) to fabricate bilayer ternary samples. Some devices prepared under other conditions will be informed in the following section. Neither the thickness adjustment nor the ratio variation improves the efficiency larger than 0.9%. A current density–voltage (J–V) curve of a bilayer ternary device is shown in Figure 2b. The efficiency of this device is 0.82%. The photovoltaic parameters of this device are listed in Table 1. This device is called device S. Our optimization procedure is similar to that in polymer bilayer ternary devices [15,16]. The present experiment shows that thermal deposition is the other approach to fabricate bilayer ternary devices rather than the solution method.

Among various attempts, we find that the interface between two binary BHJs has the most significant influence on the efficiency of the bilayer ternary devices. After the oblique deposition of ZnPc:C60 BHJ, the simplest way to deposit the next ClAlPc:C60 BHJ is to switch the evaporation source (shutting ZnPc and turning on ClAlPc quickly) while maintaining the evaporation of C60. However, it is almost impossible to complete these operations precisely and simultaneously by manual operation, additionally the substrate must be rotated to the horizontal position at the same time. In order to prepare a bilayer ternary active layer with a sharp interface, we designed a switching procedure as follows: shut both evaporation sources of ZnPc and C60 simultaneously, then rotate the substrate, and finally turn on the evaporation source of ClAlPc and C60. The above devices with the efficiency of ~0.8% are fabricated in this way. This sharp interface between ClAlPc:C60 and ZnPc:C60 BHJ looks like the interface formed by the transfer technique in polymer bilayer ternary devices, and we call this kind of interface a standard interface.

It is known that the film formed by thermal deposition can be adjusted accurately by tuning the evaporation condition. Greater efficiency in bilayer ternary devices is obtained by accurately changing the interface deposition procedure. The termination of evaporation in C60 is 10 s later than that of the ZnPc source intentionally. An additional pure C60 layer is deposited in this way. The thickness of C60 layer is ~0.5 nm, which is calculated with the evaporation rate (0.05 nm/s). Comparing to the diameter of C60 molecule (~0.7 nm), this thickness means that an approximately single monolayer of C60 molecules is covered on the ZnPc:C60 BHJ. Then, ClAlPc:C60 BHJ is deposited consequently after the substrate is rotated to the horizontal position. We call this kind of interface an improved interface. A typical J–V curve for a photovoltaic cell with the improved interface is shown in Figure 2b. This device has an efficiency of 1.14%. This device is called device I. Comparing this with device S, a higher Jsc and similar Voc and FF are obtained in device I. Obviously, the improved interface causes the enhancement of Jsc. No such performance is reported in the polymer bilayer ternary device. The optical absorption spectra of ZnPC:C60/ClAlPc:C60 bilayer with different interfaces are shown in Figure 2c, demonstrating the negligible difference at all peaks due to the thin C60 interfacial layer. Because this interfacial layer almost does not influence the optical absorption in the bilayer ternary device, there must be another effect correlated to the improved interface. The FF in the device with a standard interface (0.43) is just slightly smaller than that in the device with an improved interface (0.46), suggesting a similar degree of carrier collection in the bias range from 0 V to Voc. We extracted device factors, e.g., series resistance R_s_ and shunt resistance R_sh_, from the J–V curve according to the method reported in Ref. [16]. The values are listed in Table 1. Smaller R_s_ and similar R_sh_ are found in the device with an improved interface. Although a smaller R_s_ appears in the device with an improved interface, the actual effect of interfacial variation on Jsc is not clear yet.

Based on the structure drawn in Figure 1c, bilayer ternary active layer can be regarded as a special ternary blend, in which two donors reside on two sides of the interface while the acceptor disperses in the whole bilayer. Efficient transport of both hole and electron should be guaranteed within this model. However, Colberts et al. find a particular phenomenon in the polymer bilayer ternary device, which is five times the magnification of the external quantum efficiency (EQE) under both 730 nm light irradiation and a bias of −5 V [15]. This phenomenon is suspected due to a barrier at the interface between two BHJs, where a large number of electrons, dissociated from photo-generated excitons, are trapped. However, an intrinsic electron barrier seems to be in contrast to the continuous distribution of fullerene molecules in the BHJ bilayer. Some preliminary studies show that changing the polymer in the bottom BHJ does not eliminate this barrier behavior [15]. No investigation of the interfacial effect is conducted in previous studies, partly because of the difficulty in tuning the interface between two binary BHJs by the solution method. This barrier phenomenon remains unclear in the literature. In the present experiment, we demonstrate a clear influence of the interfacial layer on the performance of bilayer ternary cells.

The Jsc of the device with an improved interface is larger than that of devices with a standard interface. Considering the structure and thickness, the devices with and without the C60 molecular monolayer should generate almost the same number of charge carriers under the same illumination. Therefore, the charge transport across the interface should be the main reason for the difference in Jsc value between these two devices, because other parts in two devices are all the same for charge carriers. We propose that some extra charge recombination occurs at the standard interface, but such negative effect disappears in the case of the improved interface.

**Figure 4:**
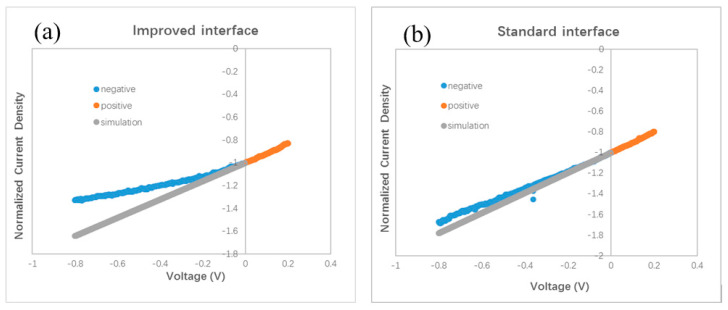

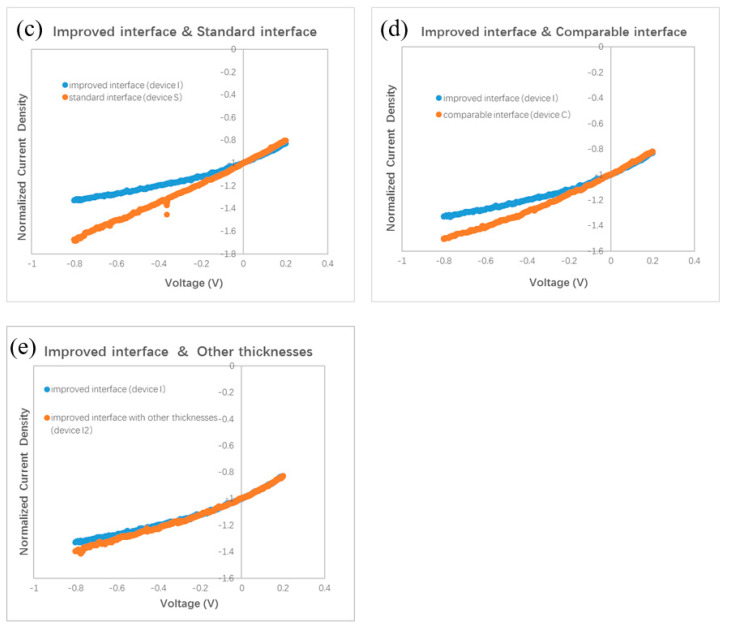
Normalized J–V curves. (**a**) with an improved interface, (**b**) with a standard interface, (**c**) device I and device S, (**d**) device I and device C, (**e**) device I and device I2.

The slope of the J–V curve has been employed to study the charge loss and trap information in organic BHJ solar cells. An oblique J–V curve crossing the Jsc point usually means some charge losses due to recombination [24]. Simulation of oblique J–V curves reveals different kinds of voltage-dependent recombination in polymer BHJ solar cells, including geminate recombination and nongeminate recombination via traps [25,26,27]. We analyze the J–V curve in different devices based on the result published in Ref. [25]. The oblique J–V curves surrounding the Jsc point in Figure 2b indicate the occurrence of voltage-dependent recombination in bilayer ternary devices. We redraw the J–V curves in Figure 4 in order to clarify the difference between the stand and improved interface. Each J–V curve is normalized to its own Jsc and divided in half at the Jsc point. The slope of the J–V curve under positive bias (0–0.2 V) is used as a benchmark for comparison with that under negative bias (−0.8–0 V). A straight grey line is drawn in Figure 4a,b by extending the J–V curve from the positive side to the negative side. Figure 4a reveals that a slight saturation trend emerges in the bilayer ternary device with an improved interface when the negative bias enlarges gradually. Such a phenomenon is common in binary BHJ devices with an oblique J–V curve under negative bias, reflecting that more charge carriers are extracted under a stronger field and that less recombination occurs inside the device [24,25,26,27]. By contrast, an almost constant slope appears in the bilayer ternary device with a standard interface under both positive and negative bias, which is shown in Figure 4b. This straight J–V curve crossing the Jsc point reveals that charge recombination in the bilayer ternary device with a standard interface is almost independent of the bias. Because these two devices are fabricated under the same conditions, except the interfacial layer between two BHJs, the different style of J–V curves under negative bias should come from the interfacial layer and indicates different degrees of charge recombination originating in the different interfaces.

A smaller Jsc has been found in a polymer BHJ device due to geminate or monomolecular recombination losses [26]. Moreover, compared to the J–V curve with only voltage-dependent geminate recombination, a larger slope can be seen in the J–V curve with severe nongeminate recombination under negative bias [25]. A lower Jsc is measured in the device with a standard interface compared with the device with an improved interface. Then we examined the J–V curves under a small bias. Figure 4c shows one part of the normalized J–V curves of the devices with different interfaces. Two curves almost overlap under the small positive bias. Nonetheless, these two curves separate and form a branch under the negative bias. This branch shows that the slope of J–V curve of the device with an improved interface decreases gradually under a larger negative bias, whereas in the device with a standard interface, the slope of the J–V curve almost remains stable under both positive and negative bias. Such a branch of the J–V curves in the negative side is similar to the shape of the J–V curves in Ref. [25], suggesting different degrees of charge losses due to nongeminate recombination at the different interfaces. Therefore, both a smaller Jsc and larger slope under negative bias in the device with a standard interface, reveal the occurrence of severe nongeminate recombination at the standard interface. Because the interfacial layer between two BHJs in a bilayer ternary device has no counterpart in a traditional binary BHJ device, no clue to how to treat this standard interface can be gained from the previous literature. In our experiment, a larger Jsc and smaller slope under negative bias, after inserting a monolayer of C60 molecules between two BHJs, indicate less interfacial traps resulting from the disappearance of interfacial mixture composing three kinds of molecules (ZnPc, ClAlPc, and C60 in our experiment). Because the thin C60 layer can hardly improve the hole transport, we think that the saturation trend in the J–V curve under negative bias hints towards a reduced number of trapped electrons at the improved interface.

Our finding of the effect of the standard interface is similar to the unsatisfied behavior of polymer bilayer ternary devices [15]. We conduct further investigations on the effect of the interface by fabricating other devices with different procedures. Different from the standard and improved interface, we intentionally turned off the evaporation source of the C60, 10 s earlier than the ZnPc. We call this kind of interface a comparable interface. The measured J–V curve is shown in Figure 2b. The efficiency of this device is 0.87%, which is similar to that with the standard interface, but lower than that with an improved interface. This device is called device C. The normalized J–V curves of device C and device I are shown in Figure 4d. It can be seen that the two curves overlap under a small positive bias, whereas under the negative bias, a branch appears. This result reveals that the optimal performance must be obtained with an interfacial C60 monolayer.

**Figure 5:**
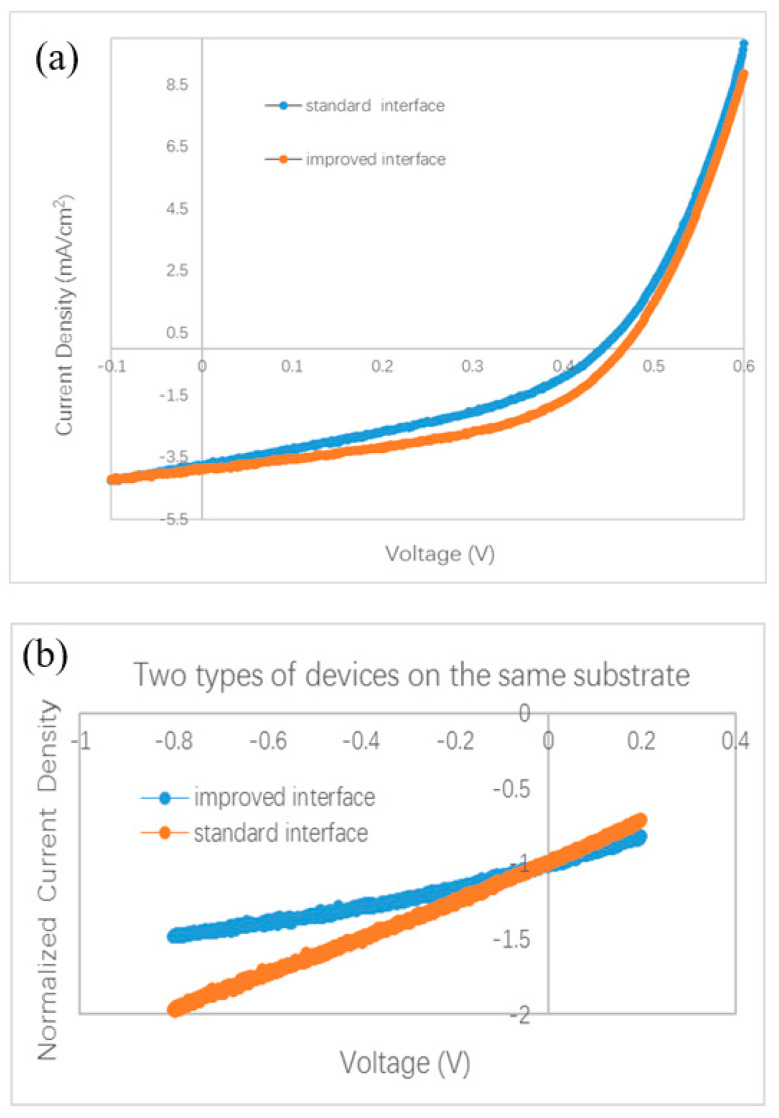
Photovoltaic behavior of bilayer ternary devices fabricated on a same substrate. Except the interfacial C60 film between ZnPc:C60 BHJ and ClAlPc:C60 BHJ, all other layers in two devices are deposited in a same deposition process. (**a**) J–V curves, (**b**) normalized J–V curves.

More experiments were performed to testify the effect of the interface between two BHJs. Because it is almost impossible to distinguish the effect of the C60 layer on a surface in air by common characterization techniques, we did not split the bilayer ternary structure to investigate the BHJ sample covered by the C60 monolayer. We designed a special experiment to check the effect of the C60 interfacial layer in completed devices. An ITO substrate was first coated by ZnPc/ZnPc:C60 bilayer. Then the sample holder was rotated to a particular position to shade part of the substrate. A thin C60 film (thickness of 1.0–1.5 nm) was deposited on only part of the substrate, while the other part of the substrate was kept away from the evaporated C60 flux. At last, the substrate was moved back to its horizontal position, and ClAlPc:C60/C60/ClAlPc/Al layers were deposited on the whole substrate. Both the standard interface and the improved interface were fabricated on the same substrate in this way, and the devices have the same thickness and microstructure in all layers except for the interfacial layer between the two BHJs. The measured and normalized J–V curves are shown in Figure 5a,b, respectively. A higher Jsc (3.87 mA/cm^2^) is also obtained in the device with an improved interface comparing to that in the device with a standard interface (3.79 mA/cm^2^). A branch is also observed in the normalized J–V curves under negative bias. Obviously, better photovoltaic performance is brought by the C60 interfacial layer. We note that because the experimental process is a little different from the optimal one, smaller efficiencies were measured in this case. This experiment definitely confirms the effect of a C60 interfacial layer between two BHJs on the efficiency and Jsc in bilayer ternary devices.

We checked the effect of the improved interface in more devices with different thickness. A J–V curve for a device with ZnPc:C60(30 nm)/ClAlPc:C60(25 nm) with an improved interface (named as device I2) is also shown in Figure 2b. The efficiency of this device is 1.04%. The normalized J–V curves of device I2 and device I are shown in Figure 4e. It can be seen that these two curves almost overlap in the whole range of both positive and negative bias. This phenomenon confirms less charge recombination in devices with an improved interface, resulting from separated ZnPc:C60 and ClAlPc:C60 BHJs. The average efficiency of the devices with an improved interface is ~1.1%, revealing a more decisive role of the interface than the thickness of a single BHJ.

It is not clear what the origin for the traps in the interfacial layer is. The sharp interface between two BHJs brings two new factors not appearing in traditional binary BHJ devices, morphological or electronic interaction at the interface. We fabricate a comparative device with the structure of ITO/ZnPc/ClAlPc:C60/C60/ClAlPc/Al, in which triple components (ZnPc, ClAlPc, and C60) meet directly in this single BHJ case. A worse photovoltaic performance is obtained in this device. More details of this device are described in the Supplementary Information (Figure S2). The J–V curve of this device shows an unchanged slope under both positive and negative bias. This phenomenon indicates that BHJ morphology, besides the interface, may not be the reason for the traps in the standard interface. We propose that other than the energy level shift, the interplay among triple components should also be considered in bilayer ternary devices in the future. This has already been discussed in ternary BHJ devices [8].

Our experiment clearly reveals the location of an additional electron barrier in bilayer ternary devices. We also show an approach to eliminate this barrier and improve the performance of bilayer ternary cells. This method can also be used to tune the interface in polymer bilayer ternary devices. In the literature of bilayer ternary devices, a two-step stacking technique is employed to assemble the bilayer ternary active layer [15,16]. If a thin C60 layer is deposited on the surface of the bottom BHJ layer, the barrier between two BHJs may also be eliminated.

## 4. Conclusions

In conclusion, small molecule bilayer ternary solar cells based on ZnPc, ClAlPc, and C60 are fabricated. The Jsc is improved by tuning the interface between ZnPc:C60 BHJ and ClAlPc:C60 BHJ. A C60 molecular monolayer is found to be beneficial for the electron transport in the bilayer ternary cell. Our result shows that the interface in bilayer ternary solar cells cannot be simply treated as a copy in binary BHJ devices.

## Figures and Tables

**Table 1:** Photovoltaic performance and extracted parameters of devices.

Device No.	Jsc (mA/cm^2^)	Voc (V)	FF	PCE	Series Resistance (R_s_) (Ω/cm^2^)	Shunt Resistance (R_sh_) (Ω/cm^2^)
Standard interface (Device S): ZnPc:C60 (30 nm)/ClAlPc:C60 (30 nm)	3.90	0.49	0.43	0.82%	3.3	266
Improved interface (Device I): ZnPc:C60 (30 nm)/ClAlPc:C60 (30 nm)	5.25	0.47	0.46	1.14%	1.4	256
Comparable interface (Device C): ZnPc:C60 (30 nm)/ClAlPc:C60 (30 nm)	4.18	0.44	0.47	0.87%	3.4	296
Improved interface (Device I2): ZnPc:C60 (30 nm)/ClAlPc:C60 (25 nm)	4.88	0.48	0.45	1.04%	3.2	288

## Supplementary Materials

The following are available online at https://www.mdpi.com/1996-1944/13/21/4833/s1. Figure S1: Schematic demonstration of co-deposition of ZnPc and C60 on ZnPc coated ITO substrate to fabricate ZnPc/ZnPc:C60 bilayer. (a) Horizontal substrate, amorphous ZnPc:C60 BHJ forms on the ZnPc film. (b) Face-on ZnPc grains grown on particularly treated ITO substrate, (c) Tilted substrate, phase separation occurs in ZnPc:C60 BHJ in this case. Blue ellipse and black dot stand for ZnPc and C60 molecule, respectively. Figure S2: Normalized J–V curve of ITO/ZnPc/ClAlPc:C60/C60/ClAlPc/Al device.
