# Supplementary material for: Interface Tuning between Two Connecting Bulk Heterojunctions in Small Molecule Bilayer Ternary Solar Cells
Source: Materials (Basel). 2020 Oct 29;13(21):4833. doi: 10.3390/ma13214833 (PMC7663015; doi:10.3390/ma13214833)
Supplement: Supplementary file 1 [file materials-13-04833-s001.pdf]

materials

Supplementary

Interface Tuning between Two Connecting Bulk Heterojunctions in Small Molecule Bilayer Ternary Solar Cells

Qi Jiang and Yingjie Xing *

Key Laboratory for the Physics and Chemistry of Nanodevices, Beijing Key Laboratory of Quantum Devices, and Department of Electronics, Peking University, Beijing 100871, China; 1701213869@pku.edu.cn
* Correspondence: xingyj@pku.edu.cn

Received: 24 August 2020; Accepted: 25 September 2020; Published: date

1. Oblique Angle Deposition Technique Used for Deposition of ZnPc/ZnPc:C60 Bilayer

Bulk heterojunction organic solar cells based on small molecules are often fabricated by co-deposition of donor and accepter materials on substrates placed horizontally. An intimate blend of donor and accepter molecules is the common product in as-prepared samples. In these bulk heterojunction photovoltaic devices, it is found that proper phase segregation in the blend film is one key factor to obtain a high power conversion efficiency, which brings less charge loss and more optical absorption. We use oblique angle deposition technique to induce phase separation in small molecule solar cells.

It has been shown that face-on ZnPc grains exhibit some capability of promoting the formation of ZnPc nuclei during the co-evaporation of ZnPc and C60, but edge-on ZnPc grains do not have such capability. [1] As a result, amorphous ZnPc:C60 BHJ forms on edge-on ZnPc grains, whereas phase separation in ZnPc:C60 BHJ occurs on face-on ZnPc grains. Because edge-on ZnPc grains always grow on ITO substrate (shown in Figure S1a), particular treatment (CuI layer) must be proceeded to ITO substrate to grow face-on ZnPc grains (shown in Figure S1b) [2].

Different from the substrate treatment, we find an alternative way to partly reproduce the face-on "surface" with edge-on ZnPc grains. When we rotate the substrate, some side facets of ZnPc grains will be forced to expose to the evaporant flux. The "surface" of ZnPc grains opposite to the evaporant flux partly looks like the surface of face-on ZnPc grains (shown in Figure S1c). In this way, the requirement for phase segregation in BHJ is introduced in our experiment without substrate treatment. The angle of the substrate relative to the incident beam is tuned in the vacuum chamber *in situ* by manual operation. The oblique angle in present experiment is—55°. [3]

Figure S1a,c show two types of deposition schematically. A thin layer of ZnPc is deposited on ITO substrate firstly. Edge-on ZnPc grains grow on both horizontal-placed and tilted ITO substrate (shown in Figure S1a,c). Then ZnPc and C60 molecules are evaporated simultaneously. The oblique substrate exposes some similar deposition facets as those in face-on ZnPc, resulting in phase segregation in ZnPc: C60 BHJ (shown in Figure 3a in the manuscript). This technique is also effective to create phase separation in another small molecule BHJ, ZnPc: 3,4,9,10-perylene tetracarboxylic bisbenzimidazole (PTCBI). [4] Better photovoltaic behavior is observed in both ZnPc: C60 BHJ and ZnPc:PTCBI BHJ devices prepared by this technique.

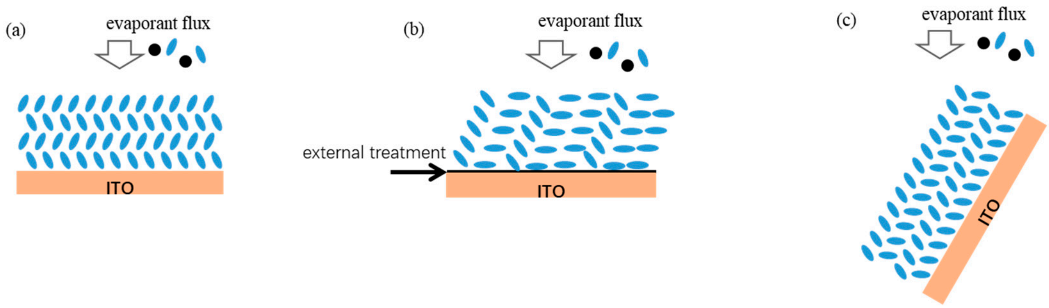

Figure 1. Schematic demonstration of co-deposition of ZnPc and C60 on ZnPc coated ITO substrate to fabricate ZnPc/ZnPc:C60 bilayer. (**a**) Horizontal substrate, amorphous ZnPc:C60 BHJ forms on the ZnPc film. (**b**) Face-on ZnPc grains grown on particularly treated ITO substrate, (**c**) Tilted substrate, phase separation occurs in ZnPc:C60 BHJ in this case. Blue ellipse and black dot stand for ZnPc and C60 molecule, respectively.

2. Demonstration of Interface Interaction in Bilayer Ternary Device

ZnPc:C60/ClAlPc:C60 bilayer ternary structure brings two new factors not occurring in traditional binary BHJ devices, morphological and/or electronic interaction at the interface. We fabricate a device in the architecture of ITO/ZnPc/ClAlPc:C60/C60/ClAlPc/Al, in which triple components (ZnPc, ClAlPc, and C60) meet directly but not in the bilayer ternary structure. Worse photovoltaic performance is obtained in this single BHJ device. Normalized J-V curve of this device shows an unchanged slope under both positive and negative bias (shown in Figure S2), indicating the occurrence of severe recombination in this device. This phenomenon indicates that BHJ morphology beside the interface may not be the reason for the traps in the standard interface.

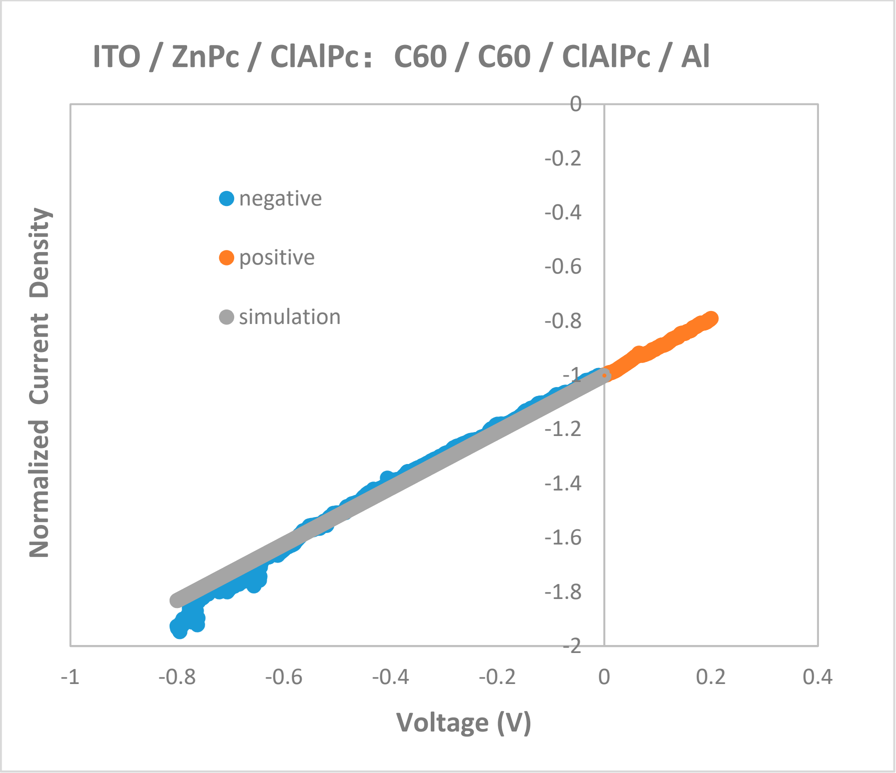

Figure 2. Normalized J-V curve of ITO/ZnPc/ClAlPc:C60/C60/ClAlPc/Al device.

References

1. Zhou Y., Taima T., Miyadera T., Yamanari T., Kitamura M., Nakatsu K. and Yoshida Y. Phase separation of co-evaporated ZnPc:C60 blend film for highly efficient organic photovoltaics. *Appl. Phys. Lett.* **2012**, *100*,233302.
2. Zhou Y, Taima T., Miyadera T., Yamanari T., Kitamura M., Nakatsu K. and Yoshida Y. Glancing Angle Deposition of Copper Iodide Nanocrystals for Efficient Organic Photovoltaics. *Nano Lett.* **2012**, *12*, 4146−4152.
3. Zhu L., Zhao T., Li K., Sun W. and Xing Y. Bulk heterojunction organic solar cells fabricated by oblique angle deposition. *Phys.Chem.Chem.Phys*. **2015**,*17*, 28765.
4. Zhao T, Zhang G. and Xing Y. Improved performance of small molecule solar cell by using oblique deposition technique and zinc phthalocyanine cathode buffer layer. *RSC Adv.* **2018**,*8*, 10999.

© 2020 by the authors. Submitted for possible open access publication under the terms and conditions of the Creative Commons Attribution (CC BY) license (http://creativecommons.org/licenses/by/4.0/).
